# A Phase II Multicenter Trial on High-Dose Vitamin D Supplementation for the Correction of Vitamin D Insufficiency in Patients with Breast Cancer Receiving Adjuvant Chemotherapy

**DOI:** 10.3390/nu13124429

**Published:** 2021-12-10

**Authors:** Elodie Chartron, Nelly Firmin, Célia Touraine, Angélique Chapelle, Eric Legouffe, Lobna Rifai, Stéphane Pouderoux, Lise Roca, Véronique D’Hondt, William Jacot

**Affiliations:** 1 Department of Medical Oncology, ICM Val d’Aurelle, Montpellier University, F-34298 Montpellier, France; Elodie.Chartron@icm.unicancer.fr (E.C.); Nelly.Firmin@icm.unicancer.fr (N.F.); Stephane.Pouderoux@icm.unicancer.fr (S.P.); Veronique.Dhondt@icm.unicancer.fr (V.D.); 2 Institut de Recherche en Cancérologie de Montpellier (IRCM) INSERM U1194, F-34298 Montpellier, France; 3 Biometrics Unit, Montpellier Cancer Institute, University of Montpellier, F-34000 Montpellier, France; celia.touraine@icm.unicancer.fr (C.T.); Lise.Roca@icm.unicancer.fr (L.R.); 4 Institut de Cancerologie du Gard, Oncogard, F-30103 Nimes, France; a.chapelle@oncogard.com (A.C.); legouffe.oncogard@orange.fr (E.L.); 5 Clinical Research Department, ICM Val d’Aurelle, Montpellier University, F-34298 Montpellier, France; Lobna.Rifai@icm.unicancer.fr

**Keywords:** vitamin D insufficiency, breast cancer, adjuvant chemotherapy, hypercalciuria

## Abstract

Breast cancer (BC) treatments induce vitamin D (VD) insufficiency and bone metabolism changes, resulting in osteoporosis and skeletal morbidity risk. We report the results of a bicentric phase II trial (ClinicalTrials.gov Identifier: NCT04091178) on the safety and efficacy of high-dose oral VD supplementation for VD deficiency correction in 44 patients with early BC treated with adjuvant chemotherapies. Patients received one dose of 100,000 IU 25-OH VD every 3 weeks from day 1 of cycle 1 to day 1 of cycle 5. The primary endpoint was the percentage of patients achieving serum 25-OH VD concentration normalization on day 1 of cycle 6 (D1C6). Secondary endpoints were safety, VD and calcium parameters at baseline and during chemotherapy, and identification of predictive biomarkers of VD normalization on D1C6. On D1C6, 21 patients (47.7%, 95% CI: 33.0–62.8) achieved VD normalization. No VD-related clinical toxicity was reported. However, 13 patients (29.5%) presented asymptomatic grade 1 hypercalciuria, leading to interruption of the high-dose oral VD supplementation in 10, followed by a rapid reduction in serum VD concentration. No baseline clinical factor was predictive of VD normalization on D1C6. This high-dose VD supplementation appears safe and efficient in patients with early BC receiving adjuvant chemotherapy.

## 1. Introduction

Therapies for early-stage and locally advanced breast cancer (BC) may induce bone loss [1]. Indeed, chemotherapy is often associated with premature ovarian failure and direct cytotoxic bone effects, resulting in bone mineral density reduction that increases the skeletal morbidity risk compared with women without BC history [1,2,3,4]. Moreover, adjuvant aromatase inhibitors, used in postmenopausal patients with BC, are associated with higher loss of bone mineral density and osteoporosis risk [5].

Vitamin D (VD) insufficiency is extremely frequent in the general population (around 50%) [6] and even more in patients with cancer. For example, 70–90% of women with early BC present VD insufficiency [7,8,9]. In BC, VD insufficiency is associated with adverse prognosis [10], higher histological grade [7], and higher BC stage [11]. In early BC (EBC), low 25-hydroxy VD (25-OH VD) serum concentration is associated with increased risk of distant recurrence and death [7]. In women with EBC receiving neoadjuvant chemotherapy, 25-OH VD level is inversely correlated with the percentage of pathological complete response, a strong prognostic factor, especially in HER2-positive (HER2^+^) and triple-negative BC [12]. This negative correlation could be partly explained by the potential anticancer effect of VD. Normal and tumor breast tissues express 1-alpha hydroxylase [13]. This enzyme hydroxylates 25-OH VD, the major circulating VD metabolite, into its biologically active form (1,25-OH VD) that interacts with the nuclear VD receptor to modulate the expression of more than 200 genes involved in various aspects of tumorigenesis. Indeed, it has been reported that VD has antiproliferative and antiangiogenetic effects, and it induces breast cancer cell differentiation and apoptosis [14,15]. Furthermore, VD can influence the response to chemotherapeutic agents. In vitro results showed that VD potentiates the antitumor activities of anthracyclines and taxanes, two cytotoxic compounds widely used in EBC adjuvant and neoadjuvant chemotherapies [16,17].

Current guidelines recommend VD daily supplementation varying from 400 to 800 IU [18] to 2000 IU [19] of VD in order to achieve a 25-OH VD serum target concentration higher than 30 ng/mL (75 nmol/L) for patients with EBC. However, clinical trials that investigated VD supplementation and cancer risk and prognosis yielded inconsistent results. VD supplementation at the recommended doses (400 IU/day) failed to reduce BC risk in the Women’s Health Initiative support study [20]. Conversely, in another randomized trial in which higher VD doses (1100 IU/day) were used, the overall cancer risk was reduced [21]. The low-dose supplementation in the first study might not have corrected VD insufficiency, and this could explain the contrasting results. Indeed, other studies showed that, in most cases, low doses of VD (<1000 IU/day) do not correct 25-OH VD concentration in patients with BC and VD insufficiency [9,22,23]. On the other hand, recent trials confirmed the safety of VD supplementation with doses > 5000 IU/day [24,25]. We previously demonstrated in a dedicated phase 3 study that, in patients with EBC and VD insufficiency receiving adjuvant chemotherapy, high-dose VD supplementation doubled the percentage of women who achieved 25-OH VD concentration normalization compared with the supplementation at the recommended dose (30% versus 12.6%) [9]. However, 70% of patients in the high-dose VD arm still presented VD insufficiency at the intervention end. These findings indicate that, to increase VD normalization rate, higher doses and optimal observance are required. Therefore, we designed a phase II trial to assess whether high-dose VD oral supplementation can correct VD insufficiency in patients with EBC treated with adjuvant chemotherapy. We report here the results of the safety and efficacy analyses.

## 2. Materials and Methods

### 2.1. Eligibility Criteria

Women, older than 18 years of age, with histologically confirmed EBC, indication for adjuvant sequential chemotherapy (six cycles of sequential anthracycline and taxanes), Eastern Cooperative Oncology Group (ECOG) performance status of 0 or 1, and confirmed VD insufficiency (defined as a serum concentration <30 ng/mL or 75 nmol/mL) were eligible for enrollment in this study. All patients provided their written informed consent before inclusion. Trastuzumab was given concurrently with taxanes, for a total duration of 1 year, in patients with HER2^+^ EBC. HER2^+^ tumors were defined as 3+ positive by immunohistochemistry (IHC) or with *HER2* gene amplification by in situ hybridization for ambiguous (2+) tumors by IHC. Tumors were defined as triple-negative when the expression of estrogen receptor and progesterone receptor was lower than 10% by IHC and *HER2* was not amplified or 0/1+ or 2+ by IHC.

Patients were excluded in case of abnormal hematologic, hepatic, or renal function, metastatic disease, personal history of another cancer treated in the previous 5 years, or contraindication to calcium or VD (hypersensitivity to VD or calcium compounds, diseases inducing hypercalcemia or hypercalciuria, calcium nephrolithiasis or tissue calcifications, and hypervitaminosis D (>75 ng/mL or 187.5 nmol/mL)). Patients with significant comorbidities (e.g., uncontrolled endocrine disease, pre-existing calcium and VD balance disorders in the last 3 years, osteopenia or osteoporosis requiring VD supplementation ≥ 1000 IU/day, and medical or psychological conditions not allowing to give informed consent), patients receiving another experimental treatment, and pregnant or nursing women were not eligible. The protocol was approved by the local Ethics Committee (Comité de Protection des Personnes Sud Méditerrannée III, reference 2013.03.06 bis, 27 May 2013) and was registered at ClinicalTrials.gov (Identifier: NCT04091178).

### 2.2. Study Design and Treatment

In this phase II, open-label, nonrandomized multicenter study, patients received one dose of 100,000 IU 25-OH VD per os every 3 weeks, for outpatient clinic chemotherapy, from day 1 of cycle 1 (D1C1) to day 1 of cycle 5 (D1C5), with a daily supplementation of 1000 mg of calcium. VD administration was stopped in case of VD serum reaching the upper normal value and clinical or biological adverse event(s) related to VD or calcium metabolism (hypercalcemia > 2.65 mmol/L; hypercalciuria above the upper limit of the norm), leading to early discontinuation of the patient’s participation. At the end of the adjuvant chemotherapy cycles, VD (100,000 IU VD quarterly) and calcium (1000 mg/day) supplementation could be continued at the investigator’s discretion.

### 2.3. Endpoints and Safety Analysis

The primary endpoint was the percentage of patients with serum VD concentration normalization on day 1 of cycle 6 (D1C6). Secondary endpoints were VD normalization rate 6, 12, 18, and 24 months after VD supplementation initiation, safety, and calcium parameters (blood calcium, phosphorus, parathyroid hormone levels, urinary calcium excretion, serum VD level), as well as creatinine and uremia at baseline and their changes during adjuvant chemotherapy, and the identification of baseline clinical factors and biomarkers that could predict VD normalization on D1C6. All patients who received at least one dose of VD were included in the safety analysis.

### 2.4. Assessments

Baseline assessments included medical history, physical examination, performance status (ECOG scale), VD and calcium supplementation in the last year, complete blood count and metabolic profile including electrolytes, hepatic and renal function, and VD and calcium parameters (corrected blood calcium, phosphorus, albumin, parathormone levels, and urinary calcium excretion). All biological samples were analyzed centrally. VD levels were measured using the DiaSorin 25-HydroxyvitaminD-125 RIA kit (DiaSorin France).

The same clinical and laboratory assessments were repeated on day 1 of each 21 day chemotherapy cycle, as well as the evaluation of VD-related side-effects and compliance. After chemotherapy completion, patients were followed (clinical and laboratory analyses) every 6 months for 2 years. Adverse events were graded according to the National Cancer Institute Common Terminology Criteria for Adverse Events (NCI-CTCAE), version 4.0.

### 2.5. Statistical Analysis

Analyses were done on the evaluable population, defined as the eligible treated patients that completed the six chemotherapy cycles.

The primary endpoint was the percentage of serum VD level normalization on D1C6. Based on a Fleming single-stage design with $p_{0}$ (largest response probability) = 30%, $p_{1}$ (smallest response probability) = 55%, *α* = 5%, and *β* = 5%, 40 patients needed to be evaluated on D1C6. The normalization goal was considered as achieved if the number of successes was at least 17. Considering a 10% rate of non-evaluable patients, it was planned to include 44 patients. As the VD insufficiency prevalence in the population with EBC was evaluated to reach 70%, 63 patients needed to be included to evaluate 40 patients with VD insufficiency on D1C6.

Baseline characteristics were described by medians and range (quantitative variables) and number of observations and percentages (qualitative variables). The number of missing values was reported, and the percentages were calculated without missing values. The primary endpoint was reported with the 95% confidence interval (CI) calculated using a logit transform. Laboratory parameter changes at each chemotherapy cycle were analyzed using linear mixed models assuming linear (or piecewise linear, if more appropriate) trajectories of the laboratory parameters for each group, defined by the VD normalization status on D1C6 (yes or no). They included fixed effects (intercept, group effect, time effect (slope), and group-by-time interaction effect) and random intercept and slope effects. The final models included only the significant effects at the 5% level. Logistic regressions were used to determine what baseline laboratory parameters and clinical factors were associated with VD concentration normalization on D1C6. A *p*-value <0.05 was considered for statistical significance. All analyses were performed using the Stata software (version 13.0).

## 3. Results

### 3.1. Patient Characteristics

**Figure 1:**
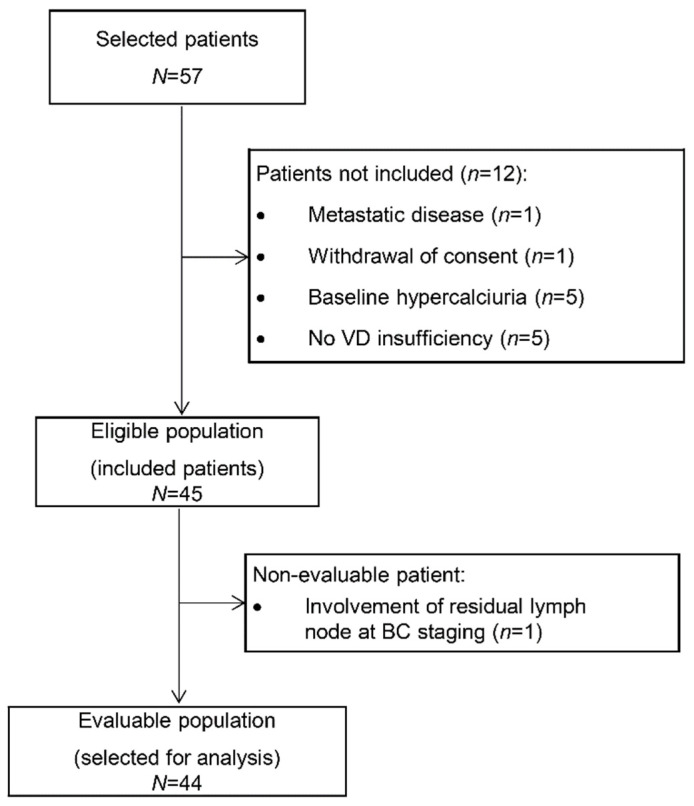
Study flowchart (VD: vitamin D).

Among the 57 patients selected at the two centers, 45 patients were finally included from June 2013 to June 2014. Twelve patients were excluded because of nonconfirmed VD insufficiency (*n* = 5), baseline hypercalciuria (*n* = 5), synchronous metastases (*n* = 1), and withdrawal of consent (*n* = 1). The primary endpoint could be evaluated in 44 patients (Figure 1).

The baseline patient characteristics are summarized in Table 1. The median age was 47.5 years, and 65.9% of patients were premenopausal. Moreover, 93% (*n* = 41) of patients had stage I or II BC, and 6.8% (*n* = 3) had stage III BC. Six tumors (13.6%) were HER2^+^, six (13.6%) were triple-negative, and 79% were hormone receptor-positive and HER2-negative. At inclusion, the median VD serum concentration was 19.15 ng/mL (range: 6.4–29.5).

Baseline calcium parameters (blood calcium, phosphorus, parathyroid hormone, and albumin levels) were normal except in five patients with low urinary calcium excretion, and two and seven patients with abnormal parathyroid hormone and phosphorus concentration, respectively (Table 2). Baseline urea and creatinine levels were in the normal range for all the included patients.

### 3.2. Treatment Compliance and Vitamin D Normalization

All patients completed the adjuvant chemotherapy treatment (D1C6) at the date of data analysis (22 February 2018), with a median follow-up of 106 weeks (30–116 weeks).

All patients took the planned VD supplementation because it was delivered at the time of outpatient chemotherapy (Supplementary Table S1). During the follow-up, the percentage of patients taking VD supplementation decreased to 65.9%, 50%, and 31.8% at months 6, 12, and 18, respectively (supplementation continued at the investigator’s discretion, Supplementary Table S2). During the chemotherapy cycles, VD intake was prematurely stopped in 10 patients due to asymptomatic hypercalciuria (safety section), while the other 34 patients (77.3%) took the VD supplementation up to D1C5. Concerning calcium supplementation, 73.8% of patients took at least 80% of the oral daily calcium supplementation on D1C1, but this percentage decreased to 59.3% on D1C6 (Supplementary Table S3).

The percentage of patients with serum 25-OH VD concentration normalization on D1C6 (primary endpoint of the study) was 47.7% (95% CI: 33.0–62.8), with a median D1C6 VD serum concentration of 29.25 ng/mL (range: 19.3–61.4). Among the first 40 evaluable patients, 18 patients (45.0%; 95% CI: 29.9–61.1) showed 25-OH VD concentration normalization on D1C6. VD normalization rates at 6, 12, 18, and 24 months were 50%, 28.9%, 80%, and 60.9%, respectively.

No baseline clinical or biological marker significantly predicted 25-OH VD concentration normalization on D1C6.

### 3.3. Toxicity

VD and calcium supplementation were well tolerated. No clinical toxicity event linked to the VD treatment was reported. However, 13 patients (29.5%) presented asymptomatic grade 1 hypercalciuria, without kidney function changes or clinical symptoms, but leading to VD supplementation interruption in 10/13 patients during adjuvant chemotherapy (D1C1 to D1C5). This interruption was rapidly followed by a reduction in VD level. The median serum VD concentration in these 13 patients was 23.1 ng/mL (range: 9.2–42.7) at hypercalciuria occurrence and 25.5 ng/mL (range: 20.1–34.6) on D1C6, and VD normalization was achieved only by three patients (23%).

### 3.4. Laboratory Parameter Changes during Adjuvant Chemotherapy

VD serum concentration significantly increased during chemotherapy. The mean increase was +3.73 ng/mL/cycle in patients with VD normalization on D1C6 and +1.78 ng/mL/cycle in patients without VD normalization.

Calcium parameter levels (corrected blood calcium, phosphorus, parathyroid hormone levels, and urinary calcium) significantly increased during adjuvant chemotherapy, while albumin levels significantly decreased. No correlation between VD serum concentration normalization status and calcium parameter changes was found.

**Figure 2:**
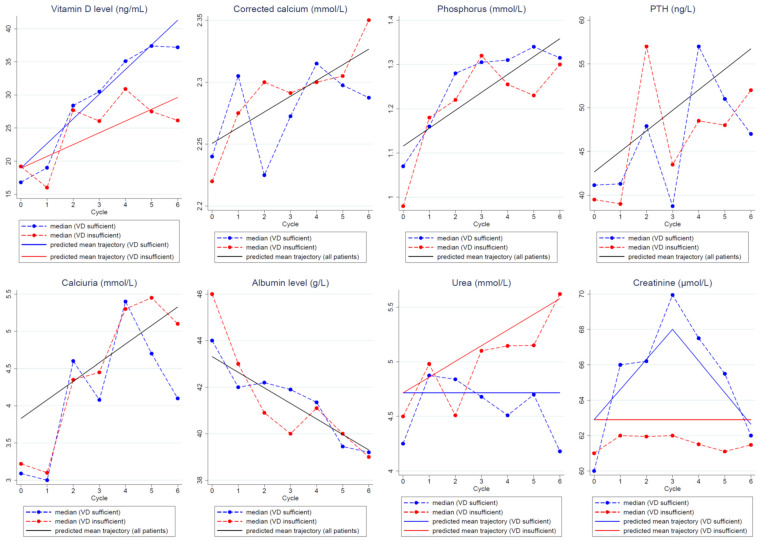
Predicted mean trajectories (solid lines) from the linear mixed models in patients with and without VD concentration normalization on D1C6 or in the whole population if the trajectories were or were not significantly different between groups, respectively. Observed median values (dashed lines) in patients with (blue) and without (red) VD concentration normalization. Abbreviations: PTH: parathormone, VD: vitamin D.

The predicted trajectories of the calcium parameters (corrected blood calcium, phosphorus, parathormone levels, and urinary calcium) and albumin during adjuvant chemotherapy were not different in patients with and without VD concentration normalization on D1C6 (Figure 2). Overall, the models predicted a significant increase in the calcium parameter levels and a significant decrease in the albumin levels during chemotherapy compared with baseline. Specifically, the models estimated a mean increase by cycle of 0.013 mmol/L for corrected calcium, of 0.040 mmol/L for phosphorus, of 2.35 ng/mL for parathyroid hormone, and of 0.25 mmol/L for urinary calcium during chemotherapy. On the other hand, uremia changes were different between patients with and without VD normalization on D1C6. Specifically, urea levels increased in patients without VD normalization on D1C6 (estimated mean increase of 0.144 mmol/L/chemotherapy cycle), while they remained stable in women with VD normalization on D1C6. Creatinine levels remained stable in patients without VD normalization on D1C6. Conversely, in patients with VD normalization, they increased up to D1C3 and then progressively decreased.

## 4. Discussion

VD insufficiency is very frequent in patients with EBC and influences prognosis. We performed a prospective phase II, open-label, nonrandomized, multicenter trial in which 44 patients with EBC received high-dose VD supplementation during adjuvant chemotherapy to increase the VD normalization rate.

The daily recommended dose of VD (400 to 800 IU of VD with 1200 mg of calcium per day) is insufficient to normalize the 25-OH VD level in most patients undergoing adjuvant chemotherapy. For instance, in a previous study, we found that serum VD concentration was normalized at adjuvant chemotherapy end in only 12% of patients when using the recommended doses of calcium and VD [9]. As VD levels have been associated with EBC prognosis in patients receiving adjuvant or neoadjuvant chemotherapy [7,12,26], VD insufficiency correction is an important issue. In the present trial, we demonstrated that high-dose VD supplementation increased the serum 25-OH VD level normalization rate on D1C6 to 47% in patients with EBC. This is better than the 30% value at 6 months we reported in a previous study in which patients with BC received a tailored high-dose VD supplementation [9].

This effect does not seem to be transient because, at month 24 of follow-up, serum 25-OH VD levels were still below the recommended levels in only 39.1% of patients with baseline VD deficiency. This value is even lower than the percentage of patients with VD insufficiency on D1C6, although only 36% of patients were still taking VD supplementation at month 24 of follow-up. This could be partly explained by previous studies showing a decrease in 25-OH VD concentration during chemotherapy and a recovery after treatment end [27,28,29]. It has been suggested that taxanes, corticosteroids, behavioral changes (such as sunlight exposure, dietary intake), and gastrointestinal side-effects play a role in this initial decrease in 25-OH VD levels [27,28,29,30]. This could explain the important 25-OH VD concentration decrease at month 12 of follow-up (only 28.9% of patients with normalized 25-OH VD level). However, this observation must be interpreted with caution due to the small size of the study population and the fact that it was not a primary outcome of this study.

The high-dose VD supplementation regimen used in our study appears safe in the context of adjuvant chemotherapy. VD levels higher than the upper normal value were not observed in any patient, and clinical adverse effects that could be associated with VD supplementation (i.e., hypercalcemia and kidney stones) were not reported, in agreement with previous studies [9,25]. For instance, Khan et al. did not detect any clinical adverse effect in women with BC taking even higher VD doses (50,000 IU/week for 12 weeks) [25]. However, in our study, 29.5% of patients presented an asymptomatic increase in urinary calcium excretion that exceeded the upper normal value. The occurrence of asymptomatic hypercalciuria could not be predicted by the baseline calcium metabolism parameters and was not associated with hypercalcemia or hypervitaminosis D when detected (the highest 25-OH VD value at hypercalciuria onset was 47.2 ng/mL). We observed a similar hypercalciuria rate (35.4%) in a previous trial [31], in which 82 patients with EBC received adjuvant chemotherapy and VD supplementation at the investigator’s discretion (recommended dose) between D1C1 and D1C6. In this previous study, hypercalciuria rates were comparable in patients with/without baseline VD insufficiency and with/without VD supplementation. In the present trial, asymptomatic hypercalciuria led to VD treatment interruption and was associated with VD insufficiency persistency in 10/13 patients at the primary objective time (D1C6), thus increasing the number of patients in which serum VD concentration was not corrected by the supplementation at the primary endpoint.

Hypercalciuria is considered a sensitive indicator of VD supplementation effect [32]. However, in our previous study, hypercalciuria rate was high, without any significant difference between patients with/without VD supplementation, and VD levels were far below the upper value at hypercalciuria detection [31]. Therefore, this high hypercalciuria incidence could be linked to a direct effect of chemotherapy, concomitant corticosteroid medications, changes in nutritional habits, or early ovarian failure [22,33,34,35,36,37]. Hypercalcemia or hypercalciuria directly induced by hypervitaminosis D is caused by increased intestinal calcium absorption and bone reabsorption [38]. However, this is a rare event, generally associated with extended daily VD intake higher than 10,000 IU and 25-OH VD serum concentrations >150 ng/mL [32]. In our trial, the highest 25-OH VD serum concentration was 61.4 ng/mL on D1C6, below the upper normal value. Unfortunately, Khan et al. did not quantify urinary calcium excretion during treatment [25].

Hypercalciuria is associated with urinary lithiasis that was not observed in our sample. This could be explained by the limited number of patients who developed hypercalciuria (*n* = 13). However, we did not record any case of urolithiasis in our previous studies (total = 277 patients) [9,31]. The association of urolithiasis and VD supplementation remains debated. A systemic review of observational studies (total = 23,228 patients) showed that 1,25-OH VD blood concentration was higher in patients with than without kidney stones [39]. This review also found that 25-OH VD concentration was higher in patients with hypercalciuria-related urolithiasis than in controls and in patients with urolithiasis and normocalciuria. Recently, a meta-analysis on 32 studies that included patients taking daily VD doses ≥2800 IU for at least 1 year did not find any increased risk of clinical adverse effects, kidney stones, hypercalcemia, or hypercalciuria [40]. Thus, the etiology of hypercalciuria and its possible complications remains to be defined. However, the causality with VD supplementation and the risk of clinically significant side-effects appears extremely low. On the other hand, VD discontinuation was often associated with VD insufficiency persistence. Thus, considering the clinical–pathological consequences of VD insufficiency, continuation of VD supplementation appears reasonable in patients with asymptomatic increase of urinary calcium clearance.

Compliance remains a critical point in oral drug efficacy, especially in conditions associated with emesis, such as chemotherapy. In our trial, only 59.3% of patients presented a satisfactory compliance, defined as a dose intensity >80% for oral daily calcium supplementation. This low rate, similar to that of our previous randomized study [9], could be linked to the tedious nature of daily intake in the context of an emetic condition. Compliance optimization appears mandatory to reach VD correction. A discontinuous regimen, associated with a medical consultation (e.g., the visit for outpatient chemotherapy), could allow increasing the compliance rate. Indeed, in our study, the VD supplementation compliance rate was 77.3% on D1C6, and the only patients who did not take VD were the 10 patients who stopped the supplementation due to asymptomatic hypercalciuria.

Lastly, one important question remains regarding the prognostic impact of VD levels in EBC patients, as studies have linked VD levels to various carcinogenetic events in breast cancer (for a review, see [41]), albeit with conflicting results in the clinical setting [42]. While the number of patients included in this trial remains too low to allow an accurate evaluation of this correlation, a survival analysis including all the patients from our three VD trials in adjuvant BC ([9,31] and the current work) is planned in order to evaluate this association.

## 5. Conclusions

High-dose VD regimen increased the percentage of serum 25-OH VD concentration normalization in patients with EBC undergoing adjuvant chemotherapy. Asymptomatic hypercalciuria was frequent, but without clinical consequences. VD interruption, due to asymptomatic hypercalciuria, was associated with a rapid decrease in VD serum concentration. This indicates that it is important to better understand the causes of hypercalciuria and to determine whether and when VD supplementation can be safely continued.

## Figures and Tables

**Table 1:** Baseline patients’ characteristics.

	*N*	%
Median age, range	47.5	29–70
Menopausal status		
Pre-menopausal	29	65.9
Post-menopausal	15	34.1
ECOG Performance Status		
0	35	79.5
1	9	20.5
Median weight (kg), range	63	47–108
Median height (cm), range	163	149–178
BMI		
<25	28	63.6
≥25	16	36.4
Histologic grade		
1	1	2.3
2	27	61.3
3	16	36.4
Perivascular invasion		
No	33	76.7
Yes	10	23.3
Missing	1	
Estrogen receptor (IHC)		
<10%	9	20.9
≥10%	34	79.1
Missing	1	
Progesterone receptor (IHC)		
<10%	11	25.6
≥10%	32	74.4
Missing	1	
HER2+ (3+ by IHC and/or *HER2* amplification)		
No	38	86.4
Yes	6	13.6
Triple-negative breast cancer		
No	38	86.4
Yes	6	13.6
Pathological staging (AJCC criteria, 7th edition)		
I	14	31.8
IIA	23	52.3
IIB	4	9.1
IIIA	3	6.8

Footnote: AJCC, American Joint Committee on Cancer Classification; BC, breast cancer; BMI, body mass index; ECOG, Eastern Cooperative Oncology Group; IHC, immunohistochemistry; WHO, World Health Organization.

**Table 2:** Baseline VD and calcium parameters.

	*N*	%	Median	Range
**Parathyroid hormone (ng/mL)**			40.5	16–89
Normal	36	94.7		
Abnormal	2	5.3		
Missing	6			
Corrected calcium * (mmol/L)			2.225	2.1–2.395
Normal	43			
Abnormal	0	100		
Missing	1			
**Urine calcium (mmol/L)**	35		3.14	0.93–6.7
Normal	5	87.5		
Abnormal	4	12.5		
Missing				
**Phosphorus (mmol/L)**			0.995	0.73–1.4
Normal	35	83.3		
Abnormal	7	16.7		
Missing	2			
**25-OH VD (ng/mL)**			19.15	6.4–29.5
Normal	0	0		
Abnormal	44	100		
Missing	0			

* Corrected calcium: calcium serum concentration corrected to the albumin level with the following formula: total calcium – (albumin level (g/L) − 40)/40).

## Data Availability

The data presented in this study are available on request from the corresponding author.

## Supplementary Materials

The following are available online at www.mdpi.com/article/10.3390/nu13124429/s1: Table S1. Compliance with VD supplementation during adjuvant chemotherapy; Table S2. Compliance with VD supplementation during follow-up; Table S3. Compliance (at least 80% of the target dose) with calcium supplementation during adjuvant chemotherapy.
