# Supplementary material for: A Phase II Multicenter Trial on High-Dose Vitamin D Supplementation for the Correction of Vitamin D Insufficiency in Patients with Breast Cancer Receiving Adjuvant Chemotherapy
Source: Nutrients. 2021 Dec 10;13(12):4429. doi: 10.3390/nu13124429 (PMC8706061; doi:10.3390/nu13124429)
Supplement: Supplementary file 1 [file nutrients-13-04429-s001.zip › nutrients-1468811-supplementary.pdf]

Supplementary data

Supplementary Table S1: VD supplementation compliance during adjuvant chemotherapy

	D1C1		D1C2		D1C3		D1C4		D1C5		D1C6	
	N	%	N	%	N	%	N	%	N	%	N	%
**VD supplementation**												
**No**	0	00	1	2.3	3	5.8	5	11.4	10	22.7	10	22.7
**Yes**	44	100.0	43	97.7	41	93.2	39	88.6	34	77.3	34	77.3

D1Cx, day 1 of chemotherapy cycle x.

Supplementary Table S2: VD supplementation compliance during the follow-up

	Month 6		Month 12		Month 18		Month 24	
	N	%	N	%	N	%	N	%
**VD supplementation**								
**No**	15	34.1	22	50.0	30	68.2	28	63.6
**Yes**	29	65.9	22	50.0	14	31.8	16	36.4

Supplementary Table S3: Compliance (at least 80% of the target dose) to calcium supplementation during adjuvant chemotherapy

	C1		C2		C3		C4		C5		C6	
	N	%	N	%	N	%	N	%	N	%	N	%
**Compliance**												
**No**	11	26.2	14	33.3	15	38.5	11	31.4	12	38.7	11	40.7
**Yes**	31	73.8	28	66.7	24	61.5	24	68.6	19	61.3	16	59.3
Missing data	*2*		*2*		*5*		*9*		*13*		*17*	
